# Link Prediction based on Quantum-Inspired Ant Colony Optimization

**DOI:** 10.1038/s41598-018-31254-3

**Published:** 2018-09-06

**Authors:** Zhiwei Cao, Yichao Zhang, Jihong Guan, Shuigeng Zhou

**Affiliations:** 1 Department of Computer Science and Technology, Tongji University, 4800 Cao’an Road, Shanghai, 201804 China; 2 Key Laboratory of Embedded System and Service Computing (Tongji University), Ministry of Education, Shanghai, 200092 China; 3 Information Security Technology Division, The Third Research Institute of Ministry of Public Security, 339 Bi Sheng Road, Shanghai, 201204 China; 4 Shanghai Key Laboratory of Intelligent Information Processing, Shanghai, 200433 China; 5 School of Computer Science, Fudan University, 220 Handan Road, Shanghai, 200433 China

## Abstract

Incomplete or partial observations of network structures pose a serious challenge to theoretical and engineering studies of real networks. To remedy the missing links in real datasets, topology-based link prediction is introduced into the studies of various networks. Due to the complexity of network structures, the accuracy and robustness of most link prediction algorithms are not satisfying enough. In this paper, we propose a quantum-inspired ant colony optimization algorithm that integrates ant colony optimization and quantum computing to predict links in networks. Extensive experiments on both synthetic and real networks show that the accuracy and robustness of the new algorithm is competitive in respect to most of the state of the art algorithms. This result suggests that the application of intelligent optimization to link prediction is promising for boosting its accuracy and robustness.

## Introduction

Many complex systems, including social, biological, information and technological systems can be well described as networks where nodes represent individuals or organizations and edges represent the relationships or interactions between them. But real-world networks are typically incomplete or inaccurate, which makes it hard to completely and precisely depict the properties of these networks. To handle this problem, researchers turn to link prediction to refine the networks before their specific studies on them^1,2^.

Link prediction in complex networks concerns the probability of establishing a link between two disconnected nodes. In the past decade, there are many applications of link prediction for various networks^3–16^. For instance, in online social networks, such as Facebook, Weibo and Twitter, link prediction is used to recommend registered users to connect with someone they know but not recognized in the network^5–7^. As a personalized service, an accurate recommendation is likely to promote its users’ loyalty^5^. In biological networks, such as protein-protein interaction networks and metabolic networks, the links are typically neither complete (high false positives) nor highly reliable (high false negative)^8–10^. Accurate link prediction based on known network structure and some specific biological information is helpful for designing targeted experiments, which may substantially reduce experimental time and cost^11–14^. In the application to monitoring the network of criminals, link prediction is used to discover the possible connections among criminals (including potential criminals), which is useful for locating some specific criminals and thus detecting and disrupting terror attacks^15,16^.

So far, topology-based link prediction algorithms can be roughly divided into three categories. The first category is based on various generating mechanisms, for instance the common neighbours (CN) algorithm^17–19^ and the cannistraci-resouce-allocation algorithm (CRA, which can be also called Cannistraci-Hebb network automata model (CH) based on local community paradigm)^20–22^. Their computational complexity is relatively low, while precision is not so satisfying. The second category is based on probabilistic models, for instance the stochastic block model (SBM)^23,24^ and the fast probability block model (FBM)^25^. Relatively, their precision is generally higher, while computational complexity is likewise higher. Thus, they are not suitable for large-scale networks. The third category is model-free, such as the structural perturbation method (SPM)^26,27^. Among them, the SBM and FBM usually perform poorly because of the issues of inference in the maximum likelihood procedure, which may fall in local optimum or in the configuration of maximum likelihood that are unrealistic. The CRA (or CH) algorithm performs the best specifically on the networks that are generated by a hyperbolic geometry because its generative mechanism (which is a local community paradigm) is associated to that geometry. The SPM algorithm is the most robust and well-performed in general regardless of the hidden generative model of the network. This may originate from its model free property, which is adaptive since it does not assume any underlying mechanism.

In this paper, we propose a quantum-inspired ant colony optimization algorithm (QACO) to predict missing links in networks, which integrates ant colony optimization and quantum computing^28^. To sum up, our contributions are three-folds. First, based on the assumption that the pheromone of artificial ants can reflect the importance of a path, we introduce a biased ant colony optimization algorithm to remedy incomplete datasets. In our algorithm, the visibility integrates the quasi-local information of nodes. Second, quantum bits and quantum logic gates are introduced into the ant colony optimization algorithm, which can effectively prevent the optimization process from falling into a local optimum. Third, extensive experiments are conducted on many benchmark networks. Our experimental results show that the accuracy and robustness of the QACO algorithm is competitive in respect to most of the state of the art algorithms.

## Results

### The QACO algorithm

#### Overview

Consider an undirected network *G* = (*V*, *E*), the corresponding complete network *G*′ is constructed by connecting all the disconnected node pairs in *G*. Let all artificial ants randomly walk in *G*′, where links and nodes are allocated some amount of pheromone. The probability that a link or node is visited by ants is proportional to its pheromone.

Assume an ant visits *n* nodes, which results in a walking path. The probability that an ant travels from node *v*_*i*_ to node *v*_*j*_ is *p*_*ij*_. The value of *p*_*ij*_ depends on the pheromone of the path, the visibility of link (*v*_*i*_, *v*_*j*_) and the quantum pheromone of *v*_*j*_. After an ant reaches its destination (say *v*_*j*_), the pheromone on the links and nodes in the path will be updated according to a certain rule. In turn, the updated pheromone on the links and nodes will affect the paths of the ants in the next iteration. Generally, the pheromone and visibility of the links will lead the ants to the globally optimal paths, since following the quantum pheromone is an effective way to avoid local optimums. Finally, the pheromone *τ*_*ij*_ and visibility *η*_*ij*_ on link (*v*_*i*_, *v*_*j*_) can, to some extent, reflect the similarity between *v*_*i*_ and *v*_*j*_.

#### Quantum Pheromone

- Quantum computation: The basic principle of quantum computation is that the relative phase and the probability amplitude of each ground state of the superposition state are not constant. The occurrence probability of each ground state varies with time, leading to the corresponding variations of the superposition state. Quantum computation is based on a device called quantum gate, which can realize a logical transformation in a certain time interval. The properties of quantum computation include interference, superposition and parallelism. In this paper, we apply quantum bits to representing the quantum pheromone of nodes and quantum rotation gate to updating quantum pheromone of ants^29,30^.
- Quantum pheromone representation: In quantum computation, |0〉 and |1〉 denote two basic states of microscopic particles. An arbitrary state of a single quantum bit can be represented by a linear combination of the two basic states. The sign |〉 is called Dirac mark, which represents an eigenstate in quantum mechanics. Each quantum bit is typically in a superposition state, which is a combination of the two eigenstates. Thus, it can be represented by |*φ*〉 = *α*|0〉 + *β*|1〉, where *α* and *β* are a pair of complex numbers, which denote the probability amplitudes of quantum states. For the quantum state |*φ*〉, the probabilities of collapsing to |0〉 and |1〉 are |*α*|^2^ and |*β*|^2^ respectively, where |*α*|^2^ + |*β*|^2^ = 1.
  A quantum bit represents two states |0〉 and |1〉. Naturally, a quantum bit of length *m* can represent 2^*m*^ different states. The probability amplitude of individual *j* with *m* quantum bits is thus defined as ${P}_{j}{=[}_{{\beta }_{1}}^{{\alpha }_{1}}{|}_{{\beta }_{2}}^{{\alpha }_{2}}{|}_{\cdots }^{\cdots }{|}_{{\beta }_{m}}^{{\alpha }_{m}}]$, where |*α*_*i*_|^2^ + |*β*_*i*_|^2^ = 1 for *i* = 1, 2, …, *m*.
  In the QACO, the quantum pheromone is represented by the quantum bit. Let the size of population be *n*. The quantum pheromone is defined as *p* = (*p*_1_, *p*_2_, *p*_3_, …, *p*_*n*_), where *p*_*j*_ (*j* = 1, 2, …, *n*) is the quantum pheromone of individual *j*^31,32^.
- Quantum pheromone updating: The core of ant colony optimization algorithm is that the ants select their paths according to the density of pheromone, which can be applied to link prediction. For each ant, the probability of choosing a node is proportional to the density of pheromone left on the node. The update of the quantum pheromone intensity of a node can be implemented by updating the rotation angle of the quantum rotating gate in QACO. Concretely, the quantum bit of a node is updated by tuning the probability amplitude of quantum bit in the quantum rotating gate. Here, the quantum rotating gate is defined as:

1 $$\begin{array}{l}[\begin{array}{c}{\alpha }_{i}^{t+1}\\ {\beta }_{i}^{t+1}\end{array}]=[\begin{array}{cc}\cos ({\theta }_{i}) & -\sin ({\theta }_{i})\\ \sin ({\theta }_{i}) & \cos ({\theta }_{i})\end{array}]\end{array}[\begin{array}{c}{\alpha }_{i}^{t}\\ {\beta }_{i}^{t}\end{array}],$$

with *i* = 1, 2, …, *m*. Here, *m* is the number of ants used, ${[{\alpha }_{i}^{t+1},{\beta }_{i}^{t+1}]}^{T}$ is the probability amplitude of the *i*-th quantum bit at the *t*-th iteration. *θ*_*i*_ is the rotation angle of the *i*-th quantum bit. Its size and orientation can be determined according to the formula *θ*_*i*_ = Δ*θ* * *sign*(*α*_*i*_ * *β*_*i*_). *sign*(*α*_*I*_ * *β*_*i*_) is a sign function. For *α*_*i*_ * *β*_*i*_ > 0, *sign*(*α*_*i*_ * *β*_*i*_) = 1; for *α*_*i*_ * *β*_*i*_ < 0, *sign*(*α*_*i*_ * *β*_*i*_) = −1; and for *α*_*i*_ * *β*_*i*_ = 0, *sign*(*α*_*i*_ * *β*_*i*_) = 0. Δ*θ* usually falls in the range of [0.01*π*, 0.08*π*]^33^.

#### Parameters setting

- Parameter initialization: At the beginning, we uniformly set the pheromone on all links to
  2 $${\tau }_{ij}=\delta ,$$
  where *δ* is a constant.
- On the other hand, we set the initial value of visibility of link (*v*_*i*_, *v*_*j*_) to
  3 $${\eta }_{ij}=\sum _{z\in {\rm{\Gamma }}(i)\cap {\rm{\Gamma }}(j)}\frac{1}{{[k(z)]}^{\iota }}+\omega \sum _{x,y\in {l}_{i\to j}}\frac{1}{{[k(x)k(y)]}^{\iota }},$$
  where *ι* is a constant, varying with the topology of network. Following the settings of previous studies^34^, we set *ω* = 0.01. Γ(*i*) and Γ(*j*) denote the sets of *i* and *j*’s neighbors, respectively. *k*_*x*_, *k*_*y*_ and *k*_*z*_ denote the degrees of *x*, *y* and *z*, respectively. *l*_*i*→*j*_ denotes the node set in the paths from node i to node j, the length of which is 3. *x* and *y* are intermediate nodes in the set *l*_*i*→*j*_.
  Finally, we set the intensity of quantum pheromone at node *v*_*j*_ to
  4 $${\mu }_{j}=\frac{1}{|{\alpha }_{j}{|}^{2}},$$
  where |*α*_*j*_|^2^ denotes the probability that the quantum state of the *j*-th quantum bit collapses to |0〉, while 1 − |*α*_*j*_|^2^ denotes the probability collapsing to |1〉.
- State transition rule: In each iteration, the transition probability that an ant *k* moves from *v*_*i*_ to *v*_*j*_ is defined as
  5 $${p}_{ij}^{k}=\frac{{[{\tau }_{ij}]}^{\lambda }{[{\eta }_{ij}]}^{\kappa }{[{\mu }_{j}]}^{\nu }}{\sum _{l}{[{\tau }_{il}]}^{\lambda }{[{\eta }_{il}]}^{\kappa }{[{\mu }_{l}]}^{\nu }},$$
  where *τ*_*ij*_ and *η*_*ij*_ are the pheromone and visibility of link (*v*_*i*_, *v*_*j*_), respectively. *μ*_*j*_ is the quantum pheromone intensity of node *v*_*j*_. The parameters *λ*, *κ* and *ν* are used to control the impact of the link pheromone, visibility and node pheromone intensity, respectively.
- Fitness function: In each iteration, an ant walks through a path of *N* nodes. The path of the *k*-th ant is denoted by *S*(*k*) = (*v*_1_, *v*_2_, *v*_3_, …, *v*_*n*_), where *v*_*i*_ ∈ *V* is the *i*-th node in the path. The fitness function is defined to evaluate each path and update the pheromone. Here, the fitness function is defined as $Q(S)=C\ast \frac{1}{n}{\sum }_{i=1}^{n}d({v}_{i})$, where *C* is a positive constant and *d*(*v*_*i*_) is the degree of node *v*_*i*_. Generally, a path with more densely-connected nodes gets a higher score.
- Pheromone updating: After each iteration, the QACO algorithm updates the pheromone of each link according to the following formula:
  6 $${\tau }_{ij}^{new}=\rho \cdot {\tau }_{ij}^{old}+{\rm{\Delta }}\tau (i,j),$$
  where Δ*τ*(*i*, *j*) is the variation of pheromone on link (*v*_*i*_, *v*_*j*_), which is defined as ${\sum }_{k=1}^{m}{\rm{\Delta }}{\tau }_{k}(i,j)$. Here, Δ*τ*_*k*_(*i*, *j*) is the pheromone released by ant *k* on link (*v*_*i*_, *v*_*j*_), *ρ* is the trajectory persistence, which is in the range of [0, 1). Obviously, Δ*τ*(*i*, *j*) is proportional to the frequency that the link is visited by ants. If we consider only the fitness *Q* in the update of pheromone, the gaps of pheromone among links may grow. In this case, the paths of ants are likely to fall into local optimums. To remedy this drawback, *β*_*k*_ is introduced to Eq. (6). In Eq. (6), Δ*τ*_*k*_(*i*, *j*) is defined as *Q*⋅(1 − *|β*_*k*_|^2^), where the probability that ant *k* selects node *v*_*j*_ is proportional to *|β*_*k*_|^2^. In this way, we can effectively prevent that the gap of the pheromone among links drastically grows. In other words, it can keep the optimizing process from falling into local optimums.
- Termination conditions and evaluations: Once the number of iterations exceeds a prespecified threshold *N*_*c*_, the optimizing process will be terminated. Finally, we can obtain a matrix *τ* + *ε* · *η*, which is normally referred to as the score matrix in some ref.^35^. To evaluate the performance of the QACO algorithm for link prediction, we will compare the precision of our algorithm to that of a number of existing algorithms in the performance evaluation section.

**Algorithm 1:**
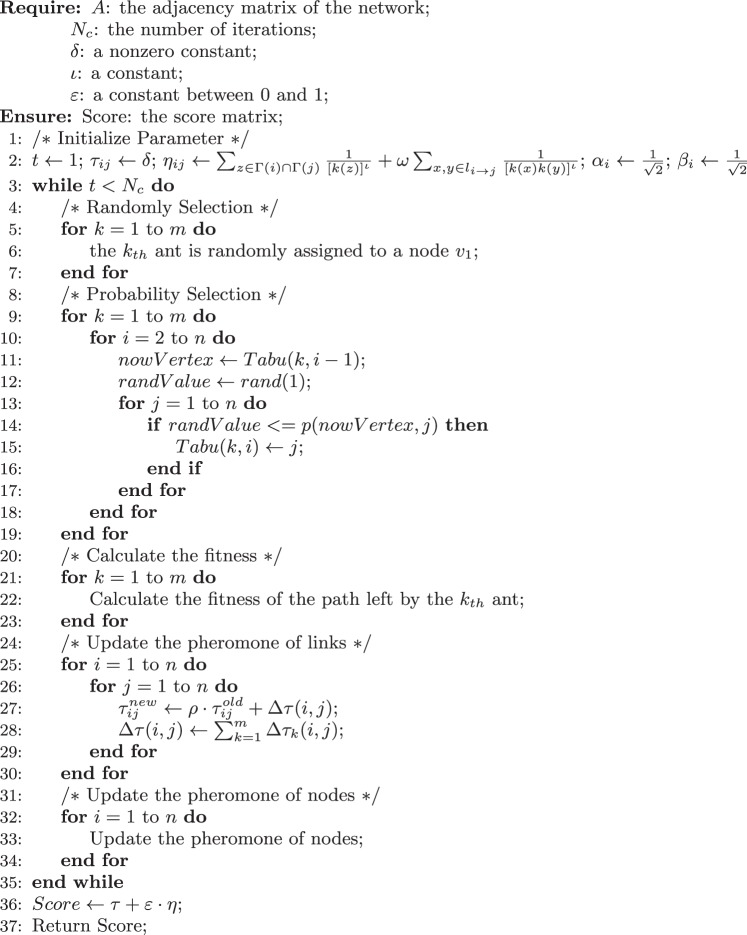
QACO for link prediction.

### The Algorithm

The proposed algorithm is outline in Algorithm 1, where the major steps are explained as follows:

1. Step 1 (Line 1–2): In the network *G* of *n* nodes, *m* ants are used. For node *j*, its status in the *t*-th iteration is $${P}_{j}^{t}=[\begin{array}{c}{\alpha }_{1}^{t}\\ {\beta }_{1}^{t}\end{array}|\begin{array}{c}{\alpha }_{2}^{t}\\ {\beta }_{2}^{t}\end{array}|\begin{array}{c}\cdots \\ \cdots \end{array}|\begin{array}{c}{\alpha }_{m}^{t}\\ {\beta }_{m}^{t}\end{array}]$$, *t* = 1, 2, …, *N*_*c*_ (the number of iterations). In the first iteration, ${\alpha }_{i}={\beta }_{i}=\frac{1}{\sqrt{2}}$ for all *i* (*i* = 1, 2, …, *m*). The initial settings of the pheromone matrix *τ* and the visibility matrix *η* follow Eq. (2) and Eq. (3), respectively. In the experiments, we set *δ*, *λ*, *κ*, *ν*, *ρ* and *ε* to 1, 1, 2, 1, 0.9 and 0.2, respectively. *ι* is determined by the topology of networks, which is set to 0 or 1.
2. Step 2 (Line 4–7): The *m* ants are randomly assigned to *m* nodes after shuffling the order of nodes. For example, to assign 3 ants to 3 of 5 nodes. We first shuffle the order of 5 nodes, say the result is 3, 1, 5, 2, 4. Then the 3 ants are assigned to the nodes of 3, 1 and 5, respectively.
3. Step 3 (Line 8–19): The transition probability of ant *k* from *v*_*i*_ to *v*_*j*_, ${p}_{ij}^{k}=\frac{{p}_{ij}^{k}}{{\sum }_{l}{p}_{il}^{k}}$. In each iteration, *m* ants simultaneously walk for *n* steps without interaction.
4. Step 4 (Line 20–34): After each iteration, the pheromone matrix *τ* of links will be updated according to Eq. (6). Similarly, the pheromone of nodes will be updated too, according to Eq. (1).
5. Step 5 (Line 36–37): Let *t* = *t* + 1. If *t* < *N*_*c*_, then go to Step 2. Otherwise, output the matrix *τ* + *ε* · *η*, which will be used to calculate the precision.

- Computational Complexity: Let *n* be the number of nodes in the network, and 〈*k*〉 be the average degree of nodes. Firstly, in order to calculate the visibility matrix *η*, all the neighbours of the second-order neighbours of each node are required to loop over. Therefore, the time complexity of calculating visibility matrix *η* is *O*(*n*〈*k*〉^3^). Secondly, the number of qubits on each node is the same as the number of ants *m*. Therefore, the time complexity of initializing quantum pheromone *μ* is *O*(*nm*). Thirdly, the paths are the traces that the ants traverse in the network. The time complexity for forming a path in each iteration is *O*(*n*^2^). Then the time complexity for recording *m* paths in *N*_*c*_ iterations is *O*(*N*_*c*_*mn*^2^). In conclusion, the overall time complexity of the QACO algorithm can be estimated as *O*(*n*〈*k*〉^3^) + *O*(*nm*) + *O*(*N*_*c*_*mn*^2^). Since 〈*k*〉, *m* and *N*_*c*_ are constants, the time complexity of the algorithm is *O*(*n*^2^).

### Experimental results and analysis

In our experiments, we provide the performance comparison of the proposed algorithm against four state-of-the-art algorithms mentioned in Section Methods, including the algorithms based on the indices of SPM, SBM, FBM and CH. Among them, the SPM, SBM and FBM algorithms are three global algorithms for topological link prediction, while the CH algorithm is a local algorithm for topological link prediction. The recent studies have confirmed that the SPM and CH algorithms are actually one of the best-performing state-of-the-art global and local algorithms, respectively^20^. In order to verify the robustness of our algorithm, many different types of networks (the artificial, small-size, large-size and time-evolving networks) introduced in Table SI of the Supplementary Information (SI) are used. In addition, considering the high time complexity of the SBM and FBM algorithms, we only compare the SPM and CH algorithms on the large-size and time-evolving networks. Their performance will be shown as the following.

#### Evaluation on artificial networks

**Figure 1:**
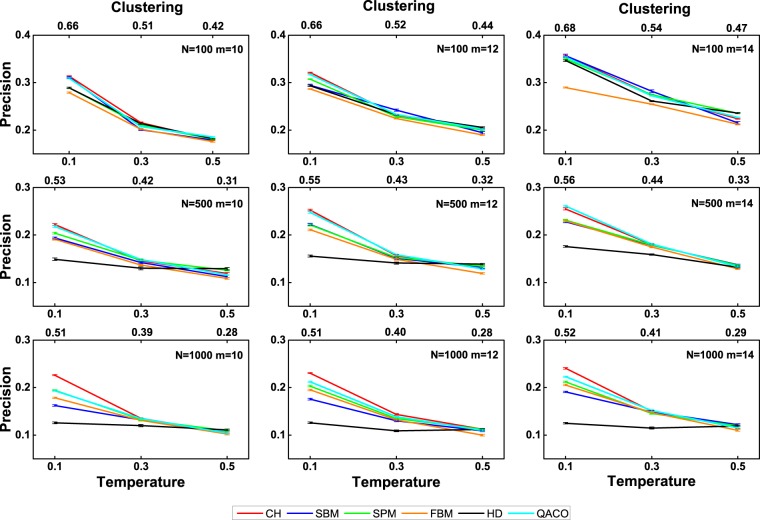
The precision evaluation of six algorithms with 90% of the links used as the training set on the nPSO networks with 8 communities. Synthetic networks are generated by the nonuniform PSO model with parameters *γ* = 3 (power-law degree distribution exponent), *m* = [10, 12, 14] (half of average degree), *T* = [0.1, 0.3, 0.5] (temperature, inversely related to the clustering coefficient), *N* = [100, 500, 1000] (network size) and 8 communities. For each combination of parameters, 100 networks are generated. For each parameter combination, the plots report the mean precision and standard error over the random iterations. Note that for SBM only 10 networks are considered due to the high time complexity. In addition, HD is the hyperbolic distances between the nodes in the original network.

**Figure 2:**
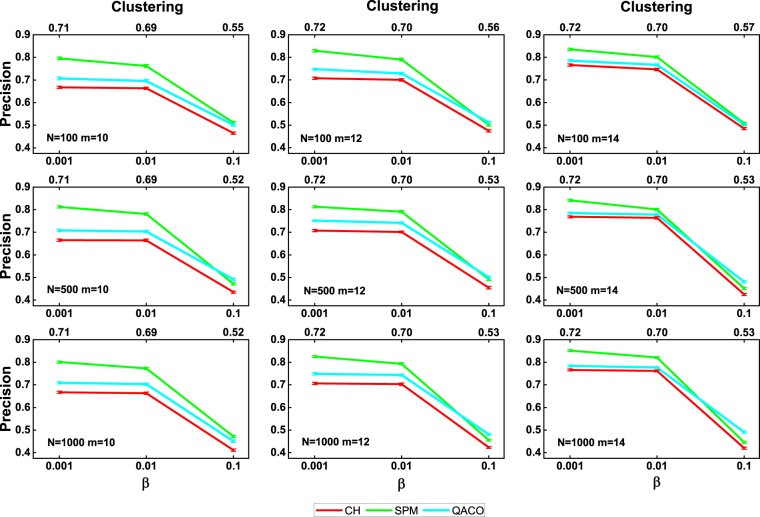
The precision evaluation of three algorithms with 90% of the links used as the training set on the Watts-Strogatz networks. Synthetic networks are generated by the Watts-Strogatz model with parameters *N* = [100, 500, 1000] (network size), *m* = [10, 12, 14] (half of average degree) and *β* = [0.001, 0.01, 0.1] (rewiring probability). For each combination of parameters, 100 networks are generated. For each parameter combination, the plots report the mean precision and standard error over the random iterations.

Figures 1 and 2 show the precision of the tested algorithms on the nPSO and WS networks, respectively. Figure 1 shows the average precision on the nPSO networks with 8 communities. We select the parameters (*r*, *m*, *T* and *N*) of the nPSO model, based on the topological features of the small-size real networks mentioned above. In Fig. 1, one can observe that the algorithms achieve fluctuating performances for low temperature (high clustering), relatively stable performances for medium temperature (medium clustering) and pretty stable performance for high temperature (low clustering). Furthermore, the performance of these algorithms generally decays with temperature. In general, the CH algorithm outperforms the other algorithms for most of the parameter domain. Meanwhile, as the network size *N* expands, the advantage of CH algorithm becomes more pronounced. Whereas, our algorithm is ranked the second. This result also confirms the robustness of our algorithm.

Figure 2 shows the average precision on the Watts-Strogatz networks. The values of *N* and *m* are the same as what they are in the nPSO networks. The values of the parameter *β* are adjusted to build a network sharing a similar clustering coefficient and average shortest path length with the nPSO networks. In Fig. 2, one can observe that the performance of the SPM algorithm is ranked first, while that of our algorithm is ranked second again. Generally speaking, the robustness of our algorithm is better than that of the SPM and CH algorithms on both artificial networks.

#### Evaluation on small-size complex networks

In order to test the robustness of our algorithm, 20 small-size network datasets are collected, which covers social networks, biological networks and technology networks, etc.

Table 1 shows the precision of the tested algorithms on the 20 small-size networks. One can observe that the SPM algorithm achieves the highest performance in 12 out of 20 networks, tying for the first place in two of them, whereas our algorithm receives the best result in four networks, tying for the first place in one of them. In Table 1, the mean precision of our algorithm over the tested datasets is tied with the SBM algorithm for second place, just following the SPM algorithm. The last row in Table 1 also shows the mean ranking of the tested algorithms on the networks. The precision-ranking metric is a more robust and reliable metric for assessing the overall performance. The mean ranking of the algorithms over all the networks represents the final evaluation score. One can observe that the SPM algorithm is still the best performing approach, as already deducible from mean precision, with an average ranking of 2.15. Our algorithm is ranked second with 2.78. The third approach is CH with 3.20. The SBM and FBM algorithms are ranked the fourth and fifth, respectively (The result of ranking score for each network can refer to Table SII in the SI). In short, compared with these state-of-the-art algorithms, the performance of our algorithm basically exceeds the CH, SBM and FBM algorithm, while lags behind the global algorithm, SPM. In addition, the performance of the CH algorithm (a local approach) exceeds that of the SBM and FBM algorithm (two global algorithms), which demonstrates its capability of prediction.

**Table 1:** The precision evaluation of five algorithms with 90% of the links used as the training set on the small-size real networks.

	*SPM*	*QACO*	*CH*	*SBM*	*FBM*
mouse neural	0.02	**0.15**	0.11	0.10	0.01
karate	0.17	0.23	0.20	**0.28**	0.27
dolphins	0.13	0.18	0.14	0.16	**0.19**
macaque neural	**0.72**	0.64	0.56	0.68	0.55
polbooks	0.17	0.17	0.17	0.15	**0.18**
ACM2009 contacts	0.26	**0.28**	0.27	0.25	0.26
football	0.31	0.30	**0.36**	0.34	0.25
physicians innovation	0.07	**0.09**	0.07	0.06	0.08
FWFW	**0.56**	0.30	0.08	0.18	0.14
manufacturing email	**0.51**	0.41	0.42	0.47	0.39
littlerock foodweb	**0.84**	0.44	0.15	0.73	0.17
jazz	**0.65**	0.48	0.56	0.47	0.45
residence hall friends	**0.28**	0.21	0.24	0.18	0.24
haggle contacts	**0.62**	**0.62**	0.57	**0.62**	0.57
worm nervoussys	**0.16**	0.13	0.12	0.15	0.11
netsci	0.41	0.37	**0.50**	0.13	0.33
infectious contacts	**0.37**	0.30	0.34	0.30	0.33
flightmap	**0.75**	0.59	0.54	0.64	0.56
email	**0.16**	0.15	**0.16**	0.09	**0.16**
polblog	**0.23**	0.20	0.17	0.19	0.17
mean precision	**0.37**	0.31	0.29	0.31	0.27
mean ranking	**2.15**	2.78	3.20	3.28	3.60

For each network, the table reports the mean precision over the random iterations and the mean precision over the entire datasets. Moreover, the mean ranking of the algorithms over all the networks is shown in the last row. In addition to 10 iterations for SBM due to its high computational time, the other algorithms are 100 iterations. For each network, the best algorithm (or algorithms) is highlighted in bold. The networks are sorted by *N* in ascending order. The algorithms are ranked from left to right according to the mean ranking (the results of ranking score for each network can refer to Table SII in the SI).

In order to check the statistical significance of the difference in performance between the algorithms, we perform the pairwise permutation tests (10,000 iterations), based on the precision-ranking values of each algorithm over the networks (columns of Table SII in the SI). Table SIII in the SI presents the pairwise p-values, adjusted for multiple hypothesis comparison by the Benjamini-Hochberg correction. The p-values lower than the significance level 0.05 are highlighted in bold. We find that the mean performances of the SPM-CH, SPM-SBM, SPM-FBM and QACO-FBM pairs are significantly different. In the sense, the result confirms the advantage of the SPM in performance, on the other hand, confirms that our algorithm is competitive in respect to the four state of the art algorithms. Note that the SPM is a global algorithm with time complexity, *N*^3^, while our algorithm is a quasi-local algorithm with time complexity, *N*^2^.

Comparing the performance of our algorithm in a variety of networks, we find that our algorithm is suitable for the networks with disassortative mixing^36^. For example, for the four networks, mouse neural, ACM2009 contacts, physicians innovation and haggle contacts networks, our algorithm is all ranked first in terms of precision-ranking. Their Pearson coefficients are −0.52, −0.12, −0.08 and −0.47, respectively. Conversely, when the Pearson coefficient is positive, our algorithm typically performs poorly. For example, the precision-ranking values of our algorithm are 4, 3, 4, 4.5 and 4 on the football, jazz, residence hall friends, infectious contacts and email networks, respectively. All of the networks are assortative mixing (The details can refer to Table SI and Table SII in the SI.).

#### Evaluation on large-size real complex networks

Aiming to test the scalability of our algorithm, 12 large-size network datasets of different type are selected, which covers social networks, internet networks and technology networks, etc. Their topological features of common interest are likewise shown in Table SI. Table 2 shows the precision of the tested algorithms on the networks. It is evident that the CH algorithm obtains the highest performance in 6 out of 12 networks, tying the first position in two of them. The overall performance can be measured by the mean ranking. The mean ranking of the CH algorithm is 1.88, which is higher than its counterparts. With respect to the value of precision, the CH algorithm performs very poorly on the router network, which drags its mean precision. This result may be induced by the sparsity of the router network (its average clustering coefficient is 0.01), which restricts the formation of local community and ultimately leads to poor performance of CH algorithm on this network. Instead, the SPM algorithm performs very well on the yeast and arxiv astroph network. The remarkable advance leads to its mean precision is ranked first. However, its mean ranking is at the bottom, since its performance on the other networks is not desirable. The performance of the QACO algorithm is ranked second in both rankings.

**Table 2:** The precision evaluation of three algorithms with 90% of the links used as the training set on the large-size real networks.

	*CH*	*QACO*	*SPM*
yeast	0.25	0.26	**0.44**
odlis	**0.12**	0.11	0.08
router	0.11	**0.33**	0.30
advogato	**0.16**	0.15	0.15
wikipedia	0.14	0.11	**0.16**
oregon	**0.08**	**0.08**	0.07
P2P	0.03	**0.04**	0.03
arxiv astroph	0.53	0.58	**0.67**
thesaurus	**0.08**	**0.08**	0.07
arxiv hepth	0.22	0.21	**0.27**
ARK201012	**0.16**	0.14	0.11
facebook	**0.11**	0.10	0.10
mean precision	0.17	0.18	**0.20**
mean ranking	**1.88**	2.00	2.13

For each network, the table reports the mean precision over the random iterations and the mean precision over the entire datasets. Moreover, the mean ranking of the algorithms over all the networks is shown in the last row. In addition, all algorithms are 10 iterations. For each network, the best algorithm (or algorithms) is highlighted in bold. The networks are sorted by *N* in ascending order. The algorithms are ranked from left to right according to the mean ranking (the results of ranking score for each network can refer to Table SIV in the SI).

We also perform the pairwise permutation tests (10,000 iterations), based on the precision-ranking values of each algorithm over the large-size real networks (columns of Table SIV). From Table SV, one can find that the mean performances of the three algorithms are not significantly different on these networks. Relatively speaking, the CH algorithm performs better on most of the large-size real networks.

#### Evaluation on time-evolving real networks

In order to maintain the diversity of evaluation framework, we adopt the evaluation framework mentioned in previous study^20^, which considers the link-growth evolution of a real network over time. Six Autonomous systems (AS) Internet topologies collected by CAIDA are selected, which is from September 2009 to December 2010, spanning 3 months in total. Their topological features of common concern are shown in Table SI.

In Table 3, one can observe that the QACO algorithm outperforms the CH and SPM algorithm, with a mean precision of 0.15. One can see that the precision grows with time, going from 0.13 to 0.16 for the QACO, from 0.11 to 0.14 for the CH and from 0.07 to 0.11 for the SPM, respectively.

**Table 3:** The precision evaluation of three algorithms in time on the AS Internet networks.

*QACO*					*CH*					*SPM*						mean precision	mean ranking
**0.13**	**0.14**	**0.15**	**0.16**	**0.16**	0.11	0.12	0.13	0.14	0.14	0.08	0.09	0.09	0.10	0.11	**QACO**	**0.15**	**1**
	**0.13**	**0.14**	**0.16**	**0.16**		0.12	0.13	0.14	0.14		0.07	0.08	0.09	0.10	CH	0.13	2
		**0.13**	**0.15**	**0.16**			0.12	0.13	0.14			0.08	0.09	0.10	SPM	0.09	3
			**0.13**	**0.15**				0.12	0.13				0.08	0.09			
				**0.14**					0.12					0.09			

From September 2009 to December 2010, six AS Internet network snapshots are considered at time steps of 3 months. For every snapshot at times *i* = [1, 5], the non-observed links are assign likelihood scores based on the algorithms. Meanwhile, the link-prediction performance is evaluated with respect to every future time point *j* = [*i* + 1, 6]. Considering a pair of time points (*i*, *j*), the non-observed links at time *i* are ranked by likelihood scores in descending order. And the precision is computed as the percentage of links that appear at time *j* among the *top* − *r* links, where *top* − *r* is the total number of non-observed links at time *i* that appear at time *j*. Non-observed links at time *i* involving nodes that disappear at time *j* are not considered in the ranking. For each algorithm, a 5-dimensional upper triangular matrix is shown, where entry (*i*, *j*) denotes the precision of the link prediction from time *i* to time *j* + 1. On the right side, the algorithms are ranked by the mean precision computed over all the time combinations. For each comparison, the best algorithm is highlighted in bold.

In conclusion, different from the removal and re-prediction framework in which the set of missing links is artificially generated by a random procedure, here the set of links that will appear after two consecutive time points is given by ground-truth information, which makes the result even more truthful and significant, confirming the effectiveness of the QACO algorithm.

In summary, our algorithm is competitive in respect to most of the state-of-the-art algorithms in balancing precision and robustness on the tested networks.

## Discussions

Inspired by ant colony optimization and quantum computing, we propose a quantum-inspired ant colony optimization algorithm for link prediction in networks. By utilizing visibility, the algorithm integrates the quasi-local structural information of individuals. By using quantum bits and quantum logic gates, it can effectively keep the optimization process from being trapped in local optimums. Compared with a series of the state-of-the-art algorithms on the artificial, small-size, large-size and time-evolving networks, our algorithm exhibits a satisfying robustness and scalability. Especially on the time-evolving real networks, our algorithm outperforms all the tested algorithms. We believe that the quantum-inspired ant colony optimization algorithm provides a new paradigm for the future studies of link prediction.

Admittedly, there may be other definitions of pheromone and visibility that can improve the performance of our algorithm. The pheromone updating strategy and the parameter selecting procedure can also be further optimized. Apart from the ant colony optimization algorithm, other intelligent optimization algorithms may be more effective on some networks. All these will be explored in our future work.

## Methods

### Link prediction problem

Consider an undirected network *G*(*V*, *E*) where *V* is a set of nodes and *E* is a set of links. Here, self-connections and multiple links are not considered. For each pair of disconnected nodes *x*, *y* ∈ *V* in the network, a link prediction algorithm assigns a score *S*_*xy*_, which indicates the probability of *x* and *y* connecting with each other. By sorting the scores of all disconnected node pairs, those pairs at the top of the list are more likely to be connected.

In order to test the performance of the algorithm, the existing links in the network, *E*, are randomly divided into two sets: the training set *E*^*T*^ and the probe set *E*^*P*^. Here, *E* = *E*^*T*^ ∪ *E*^*P*^ and *E*^*T*^ ∩ *E*^*P*^ = ∅. The algorithm estimates the scores of disconnected node pairs in *G* based on the information of the training set, and *E*^*P*^ is used as the benchmark for evaluating the prediction result.

### Evaluation metrics

In order to measure the accuracy of link prediction, we use the following metrics: Precision and Precision-ranking^37–40^.

- Precision refers to the fraction of correctly predicted links in the predicted links. It is defined as:

7 $$Precision=\frac{m}{L},$$

where *m* denotes the number of correctly predicted links, *L* denotes the number of predicted links. For a given *L*, the greater precision is, the better the performance of an algorithm is.

- Precision-ranking is a more robust and reliable metric for assessing the overall performance. For each network, all the algorithms are ranked by precision in descending order. The mean ranking of the algorithms over all the networks represents the final score.

### Benchmark algorithms

For a comprehensive comparison, three global algorithms (SPM, SBM and FBM) and a local algorithm (CH) are considered.

Let *x* and *y* be two randomly selected nodes in a network. Γ(*X*) and Γ(*y*) denote the sets of *x* and *y*’s neighbors, respectively. In the following, we will briefly introduce the definitions of the algorithms mentioned above.

The SPM algorithm is a structural perturbation method that relies on a theory similar to the first-order perturbation in quantum mechanics. The idea behind this algorithm is that a missing part of the network is predictable if it does not significantly change the structural features of the observable part^26,41^. It is thus defined as:

8 $${S}_{xy}^{SPM}=\sum _{k=1}^{N}({\lambda }_{k}+{\rm{\Delta }}{\lambda }_{k}){x}_{k}{x}_{k}^{T},$$

where *λ*_*k*_, *x*_*k*_ and Δ*λ*_*k*_ are the eigenvalue of the observed matrix, the corresponding orthogonal normalized eigenvector and the eigenvalue of a perturbation set, respectively.

The SBM algorithm is based on the assumption that the probability that two nodes are connected depends only on the groups to which they belong, and it is one of the most general network models^23^.

The FBM algorithm is a global algorithm based on the same network partitioning theory as the SBM algorithm, but it introduces a greedy strategy for an efficient sampling over the space of the possible partitions, which leads to high improvements in the computational time^25^.

The CH index is based on the assumption that two nodes are more likely to be connected if their common neighbours are densely connected^20,42^. It is thus defined as:

9 $${S}_{xy}^{CH}=\sum _{z\in {\rm{\Gamma }}(x)\cap {\rm{\Gamma }}(y)}\frac{|\gamma (z)|}{|{\rm{\Gamma }}(z)|},$$

where *γ*(*z*) refers to the sub-set of *z*′*s* neighbours which are also common neighbours of *x* and *y*. |*γ*(*z*)| is defined as the local community degree of *z*.

### Data sets

In order to validate the proposed algorithm, we provide the performance comparison over two groups of network datasets. The first group composes of two synthetic networks that are Watts-Strogatz (WS)’s small-world network^43^ and Cannistraci’s nonuniform popularity-similarity optimization (nPSO) network^44^. The WS small-world network is generated by rewiring *r* * |*E*| links on a regular lattice with *n* nodes, where *r* denotes the randomized rewiring probability and |*E*| denotes the total number of links^45,46^. The nPSO model generates synthetic networks in the hyperbolic space where heterogeneous angular node attractiveness is forced by sampling the angular coordinates from a tailored nonuniform probability distribution, and the nPSO model allows to explicitly control the size, the mixing property and the number of communities of the generated network^47^.

The second group composes of three types of real-world networks: 20 small-size, 12 large-size and 6 time-evolving real networks, which are described in detail in the SI. In addition, all real networks have been transformed into undirected, unweighted and no self-loops. Moreover, we consider only the largest component of each of the all real-world networks. The basic topological properties of the largest component in each tested network are shown in Table SI.

## Electronic supplementary material

Supplementary Information
