# Supplementary material for: Link Prediction based on Quantum-Inspired Ant Colony Optimization
Source: Sci Rep. 2018 Sep 6;8:13389. doi: 10.1038/s41598-018-31254-3 (PMC6127200; doi:10.1038/s41598-018-31254-3)
Supplement: Supplementary file 1 — Supplementary Information [file 41598_2018_31254_MOESM1_ESM.pdf]

Link Prediction based on Quantum-Inspired Ant Colony Optimization Supplementary Information

Zhiwei Cao[1,2*], Yichao Zhang[1*], Jihong Guan[1,†] and Shuigeng Zhou[3]
[1] *Department of Computer Science and Technology, Tongji University, 4800 Cao'an Road, Shanghai 201804, China*
Key Laboratory of Embedded System and Service Computing (Tongji University), Ministry of Education, Shanghai 200092, China
[2] *Information Security Technology Division, The Third Research Institute of Ministry of Public Security, Shanghai 201204, China and*
[3] *Shanghai Key Laboratory of Intelligent Information Processing, Shanghai 200433, China*
School of Computer Science, Fudan University, 220 Handan Road, Shanghai 200433, China

Data sets.

In this section, we will describe the three types of real-world networks in detail. The basic topological properties of common concern in each tested network are shown in TABLE I.

Small-size real networks.

1. "Mouse neural" is a network of synaptic connections between neurons [1].
2. "Karate" is a social network of karate club, which is collected by Wayne Zachary [2].
3. "Dolphins" is a social network of bottlenose dolphins [3].
4. "Macaque neural" is a network of macaque cortical connectome [4].
5. "Polbooks" is a network of books about US politics published during the 2004 presidential election [5].
6. "ACM2009 contacts" is a network of face-to-face contacts of the attendees of the ACM Hypertext 2009 conference [6].
7. "Football" is a network of American football games consisting of Division IA colleges during the regular season, Fall in 2000 [7].
8. "Physicians innovation" is a network for capturing innovation spread among physicians [8].
9. "FWFW" is a food web of Florida Gulf coast in the rainy season [9].
10. "Manufacturing email" is an internal email communication network between employees of a mid-sized manufacturing company [10].
11. "Littlerock foodweb" is a food web of Little Rock Lake [11].
12. "Jazz" is a collaboration network between Jazz musicians [12].
13. "Residence hall friends" is a friendship network between 217 residents living at a residence hall [13].
14. "Haggle contacts" represents the contacts between people measured by the wireless devices [14].
15. "Worm nervoussys" is a neural network of nematodes [15].
16. "Netsci" is a coauthorship network of scientists working on network theory and experiment [16].
17. "Infectious contacts" ia a network of face-to-face contacts of people [6].

---

* Equal contributors
† Electronic address: jhguan@tongji.edu.cn

18. "Flightmap" is a network of flights between Canadian and American cities [17].
19. "Email" is an email communication network in the south of Catalonia in Spain [18].
20. "Polblog" is a network of hyperlinks between weblogs on US politics [19].

Large-size real networks.

1. "Yeast" is a network of protein-protein interactions [20].
2. "Odlis" is a network of online dictionary of library and information science [21].
3. "Router" is a subnet of the internet at the router level [22].
4. "Advogato" is an online community platform for developers of free software launched in 1999 [23].
5. "Wikipedia" is a network including all the Wikipedia voting data from the inception of Wikipedia till January 2008 [24].
6. "Oregon" represents Autonomous Systems (AS) peering information inferred from Oregon route-views between March 31 2001 and May 26 2001 [25].
7. "P2P" is a sequence of snapshots of the Gnutella peer-to-peer file sharing network from August 2002 [25].
8. "Arxiv astroph" is a collaboration network of authors of scientific papers from the arXivs Astrophysics (astroph) section [26].
9. "Thesaurus" is a network of Edinburgh Associative Thesaurus [27].
10. "Arxiv hepth" is a network of publications in the arXivs High Energy Physics C Theory (hep-th) section [26].
11. "Facebook" is a network of a small subset of posts to other user's wall on Facebook [28].

Time-evolving real networks.

1. "ARK200909-ARK201012" are six Autonomous Systems (AS) Internet network snapshots, which are available from September 2009 to December 2010, at time steps of 3 months. The connections in the topology are not physical but logical, representing AS relationships [29].

Most of the real networks mentioned above can be downloaded from the Koblenz Network Collection at http://konect.uni-koblenz.de.

---

[1] Bock, D. D., Lee, W. C. A., Kerlin, A. M. et al. Network anatomy and in vivo physiology of visual cortical neurons. *Nature* **471**, 177-182 (2011).

[2] Zachary, W. W. An Information Flow Model for Conflict and Fission in Small Groups. *J. Anthropol. Res.* **33**, 452-473 (1977).

[3] Lusseau, D., Schneider, K., Boisseau, O. J. et al. The bottlenose dolphin community of doubtful sound features a large proportion of long-lasting associations: Can geographic isolation explain this unique trait?. *Behav. Ecol. Sociobiol.* **54**, 396-405 (2003).

[4] Kötter, R. Online Retrieval, Processing, and Visualization of Primate Connectivity Data From the CoCoMac Database. *Neuroinformatics* **2**, 127-144 (2004).

[5] Muscoloni, A. & Cannistraci, C. V. Local-ring network automata and the impact of hyperbolic geometry in complex network link-prediction. *arXiv preprint* arXiv:1707.09496 (2017).

[6] Isella, L., Stehlé, J., Barrat, A. et al. Whats in a crowd? Analysis of face-to-face behavioral networks. *J. Theor. Biol.* **271**, 166-180 (2011).

[7] Girvan, M. & Newman, M. E. J. Community Structure in Social and Biological Networks. *Proceedings of the National Academy of Sciences of the United States of America* **99**, 7821-7826 (2002).

[8] Coleman, J., Katz, E. & Menzel, H. The diffusion of an innovation among physicians. *Sociometry* **20**, 253-270 (1957).

[9] Ulanowicz, R. E., Bondavalli, C. & Egnotovich, M. S. Network Analysis of Trophic Dynamics in South Florida Ecosystem, FY 97: The Florida Bay Ecosystem. *Technical report, CBL* (1998).

[10] Michalski, R., Palus, S. & Kazienko, P. Matching Organizational Structure and Social Network Extracted from Email Communication. *Bus. Inf. Syst.* **87**, 197-206 (2011).

[11] Martinez, N. D. Artifacts or attributes? Effects of resolution on the Little Rock Lake food web. *Ecol. Monogr.* **61**, 367-392 (1991).
[12] Geiser, P. M. & Danon, L. Community structure in jazz. *Adv. Complex Syst.* **6**, 565-573 (2003).
[13] Freeman, L. C., Webster, C. M. & Kirke, D. M. Exploring social structure using dynamic three-dimensional color images. *Soc. Networks* **20**, 109-118 (1998).
[14] Chaintreau, A., Hui, P., Crowcroft, J. et al. Impact of human mobility on opportunistic forwarding algorithms. *IEEE Transactions on Mobile Computing* **6**, 606-620 (2007).
[15] Watts, D. J. & Strogatz, S. H. Collective dynamics of small-world networks. *Nature* **393**, 440-442 (1998).
[16] Newman, M. E. J. Finding community structure in networks using the eigenvectors of matrices. *Phys. Rev. E - Stat. Nonlinear, Soft Matter Phys.* **74**, 036104 (2006).
[17] Dueck, D. & Frey, B. J. Clustering by Passing Messages Between Data Points. *Science* **315**, 972-976 (2007).
[18] Guimerà, R., Danon, L., Díaz-Guilera, A. et al. Self-similar community structure in a network of human interactions. *Phys. Rev. E - Stat. Nonlinear Soft Matter Phys.* **68**, 1-4 (2003).
[19] Adamic, L. A. & Glance, N. The Political Blogosphere and the 2004 U.S. Election: Divided They Blog. *Proceedings of the 3rd International Workshop on Link Discovery* **417**, 36-43 (2005).
[20] Cannistraci, C. V., Alanis-Lobato, G. & Ravasi, T. Minimum curvilinearity to enhance topological prediction of protein interactions by network embedding. *Bioinformatics* **29**, 199-209 (2013).
[21] Reitz, J. M. Online Dictionary for Library and Information Science. (2002).
[22] Martinez, V., Berzal, F. & Cubero, J. C. Adaptive degree penalization for link prediction. *Journal of Computational Science* **13**, 1-9 (2016).
[23] Massa, P., Salvetti, M. & Tomasoni, D. Bowling alone and trust decline in social network sites. *IEEE International Symposium on Dependable, Autonomic and Secure Computing, DASC 2009* 658-663 (2009).
[24] Leskovec, J., Huttenlocher, D. & Kleinberg, J. Predicting Positive and Negative Links in Online Social Networks. *Proceedings of the 19th international conference on world wide web* 641-650 (2010).
[25] Leskovec, J., Kleinberg, J. & Faloutsos, C. Graphs over Time: Densification Laws, Shrinking Diameters and Possible Explanations. *ACM SIGKDD International Conference on Knowledge Discovery and Data Minin (KDD)* 177-187 (2005).
[26] Leskovec, J., Kleinberg, J. & Faloutsos, C. Graph evolution: Densification and Shrinking Diameters. *ACM Trans. Knowl. Discov. Data* **1**, 2-41 (2007).
[27] Kiss, G. R., Armstrong, C., Milroy, R. et al. An associative thesaurus of English and its computer analysis. *The computer and literary studies, A. J. Aitkin, R. W. Bailey, and N. Hamilton-Smith, Eds. Edinburgh, UK: University Press* (1973).
[28] Viswanath, B., Mislove, A., Cha, M. et al. On the Evolution of User Interaction in Facebook. *Proceedings of the 2nd ACM workshop on online social networks* 37-42 (2009).
[29] Claffy, K., Hyun, Y., Keys, K. et al. Internet mapping: From art to science. *2009 Cybersecurity Applications and Technology Conference for Homeland Security* 205-211 (2009).

TABLE I: The basic topological features of the tested small-size, large-size and time-evolving real datasets. N and $|E|$ are the total numbers of nodes and links, respectively. $\langle k \rangle$ denotes the average degree. $\langle d \rangle$ denotes the average shortest path length among all the node pairs. C denotes the average clustering coefficient. r denotes the Pearson coefficient. H denotes the degree heterogeneity, defined as $H = \frac{\langle k^2 \rangle}{\langle k \rangle^2}$.

	N	$\|E\|$	$\langle k \rangle$	$\langle d \rangle$	C	r	H
mouse neural	18	37	4.11	1.97	0.22	-0.52	1.29
karate	34	78	4.59	2.41	0.57	-0.48	1.69
dolphins	62	159	5.13	3.36	0.26	-0.04	1.33
macaque neural	94	1515	32.23	1.77	0.77	-0.15	1.24
polbooks	105	441	8.40	3.08	0.49	-0.13	1.42
ACM2009 contacts	113	2196	38.87	1.66	0.53	-0.12	1.22
football	115	613	10.66	2.51	0.40	0.16	1.01
physicians innovation	117	465	7.95	2.59	0.22	-0.08	1.25
FWFW	128	2075	32.42	1.78	0.34	-0.11	1.24
manufacturing email	167	3250	38.92	1.97	0.59	-0.30	1.66
littlerock foodweb	183	2434	26.60	2.15	0.32	-0.27	1.61
jazz	198	2742	27.70	2.24	0.62	0.02	1.40
residence hall friends	217	1839	16.95	2.39	0.36	0.10	1.21
haggle contacts	274	2124	15.50	2.42	0.63	-0.47	3.66
worm nervoussys	297	2148	14.46	2.46	0.29	-0.16	1.80
netsci	379	914	4.82	6.04	0.74	-0.08	1.66
infectious contacts	410	2765	13.49	3.63	0.46	0.23	1.39
flightmap	456	37947	166.43	1.64	0.81	-0.39	1.44
email	1133	5451	9.62	3.61	0.22	0.08	1.94
polblog	1222	16714	27.36	2.74	0.32	-0.22	2.97
yeast	2375	11693	9.85	5.10	0.31	0.45	3.48
odlis	2898	16376	11.30	3.17	0.30	-0.17	6.32
router	5021	6257	2.49	6.45	0.01	-0.14	5.50
advogato	5042	39227	15.56	3.27	0.25	-0.10	5.30
wikipedia	7066	100736	28.51	3.25	0.14	-0.08	5.10
oregon	10670	22002	4.12	3.64	0.30	-0.19	61.15
P2P	10876	39994	7.36	4.64	0.01	-0.01	1.90
arxiv astroph	17903	196972	22.00	4.19	0.63	0.20	2.99
thesaurus	23132	297094	25.69	3.49	0.09	-0.05	4.02
arxiv hepth	27400	352021	25.69	4.28	0.31	-0.03	4.14
facebook	43953	182384	8.30	5.60	0.11	0.22	2.98
ARK200909	24091	59531	4.94	3.53	0.36	-0.19	60.21
ARK200912	25910	63435	4.90	3.54	0.36	-0.18	63.00
ARK201003	26307	66089	5.02	3.53	0.37	-0.18	62.82
ARK201006	26756	68150	5.09	3.51	0.37	-0.18	62.76
ARK201009	28353	73722	5.20	3.52	0.37	-0.18	63.22
ARK201012	29333	78054	5.32	3.50	0.38	-0.18	64.96

TABLE II: The precision-ranking evaluation of the tested algorithms on the small-size real networks by precision. The mean ranking of the algorithms over all the networks represents the final evaluation for a proper comparison of the performance. For each network, the best algorithm (or algorithms) is highlighted in bold. The networks are sorted by N in ascending order.

	SPM	*QACO*	*CH*	*SBM*	*FBM*
mouse neural	4	**1**	2	3	5
karate	5	3	4	**1**	2
dolphins	5	2	4	3	**1**
macaque neural	**1**	3	4	2	5
polbooks	3	3	3	5	**1**
ACM2009 contacts	3.5	**1**	2	5	3.5
football	3	4	**1**	2	5
physicians innovation	3.5	**1**	3.5	5	2
FWFW	**1**	2	5	3	4
manufacturing email	**1**	4	3	2	5
littlerock foodweb	**1**	3	5	2	4
jazz	**1**	3	2	4	5
residence hall friends	**1**	4	2.5	5	2.5
haggle contacts	**2**	**2**	4.5	**2**	4.5
worm nervoussys	**1**	3	4	2	5
netsci	2	3	**1**	5	4
infectious contacts	**1**	4.5	2	4.5	3
flightmap	**1**	3	5	2	4
email	**2**	4	**2**	5	**2**
polblog	**1**	2	4.5	3	4.5
mean ranking	**2.15**	2.78	3.20	3.28	3.60

TABLE III: The permutation test for precision-ranking on the small-size real networks. For each pair of methods, a permutation test is the mean of 10,000 iterations, which is based on the two vectors of precision-rankings on the small-size real networks (columns of TABLE II). The table shows the pairwise p-values, adjusted for multiple hypothesis comparison by the Benjamini−Hochberg correction. The p-values lower than the significance level of 0.05 are highlighted in bold.

p-value	*SPM*	*QACO*	*CH*	*SBM*	*FBM*
SPM		0.083	**0.025**	**0.018**	**0.007**
QACO	0.083		0.157	0.130	**0.039**
CH	**0.025**	0.157		0.478	0.205
SBM	**0.018**	0.130	0.478		0.262
FBM	**0.007**	**0.039**	0.205	0.262	

TABLE IV: The precision-ranking evaluation of the tested algorithms on the large-size real networks by precision. The mean ranking of the algorithms over all the networks represents the final evaluation for a proper comparison of the performance. For each network, the best algorithm (or algorithms) is highlighted in bold. The networks are sorted by N in ascending order.

	CH	*QACO*	*SPM*
yeast	3	2	**1**
odlis	**1**	2	3
router	3	**1**	2
advogato	**1**	2.5	2.5
wikipedia	2	3	**1**
oregon	**1.5**	**1.5**	3
P2P	2.5	**1**	2.5
arxiv astroph	3	2	**1**
thesaurus	**1.5**	**1.5**	3
arxiv hepth	2	3	**1**
ARK201012	**1**	2	3
facebook	**1**	2.5	2.5
mean ranking	**1.88**	2.00	2.13

TABLE V: The permutation test for precision-ranking on the large-size real networks. For each pair of methods, a permutation test is the mean of 10,000 iterations, which is based on the two vectors of precision-rankings on the large-size real networks (columns of TABLE IV). The table shows the pairwise p-values, adjusted for multiple hypothesis comparison by the Benjamini−Hochberg correction.

p-value	*CH*	*QACO*	*SPM*
CH		0.328	0.231
QACO	0.328		0.336
SPM	0.231	0.336	
